# “He’s the Number One Thing in My World”: Application of the PRECEDE-PROCEED Model to Explore Child Car Seat Use in a Regional Community in New South Wales

**DOI:** 10.3390/ijerph14101206

**Published:** 2017-10-10

**Authors:** Kate Hunter, Lisa Keay, Kathleen Clapham, Julie Brown, Lynne E. Bilston, Marilyn Lyford, Celeste Gilbert, Rebecca Q. Ivers

**Affiliations:** 1 The George Institute for Global Health, University of New South Wales, Sydney, NSW 2052, Australia; lkeay@georgeinstitute.org.au (L.K.); mlyford@georgeinstitute.org.au (M.L.); cpoulton@georgeinstitute.org.au (C.G.); rivers@georgeinstitute.org.au (R.Q.I.); 2 Australian Health Services Research Institute, University of Wollongong, Wollongong, NSW 2522, Australia; kclapham@uow.edu.au; 3 Neuroscience Research Australia and University of New South Wales, NSW 2052, Sydney; j.brown@neura.edu.au (J.B.); L.Bilston@neura.edu.au (L.E.B.); 4 Prince of Wales Clinical School, University of New South Wales, Sydney, NSW 2052, Australia; 5 Sydney School of Public Health, The University of Sydney, Sydney, NSW 2006, Australia

**Keywords:** Aboriginal, child, car seats

## Abstract

We explored the factors influencing the use of age-appropriate car seats in a community with a high proportion of Aboriginal families in regional New South Wales. We conducted a survey and three focus groups with parents of children aged 3–5 years enrolled at three early learning centres on the Australian south-east coast. Survey data were triangulated with qualitative data from focus groups and analysed using the PRECEDE-PROCEED conceptual framework. Of the 133 eligible families, 97 (73%) parents completed the survey including 31% of parents who reported their children were Aboriginal. Use of age-appropriate car seats was reported by 80 (83%) of the participants, and awareness of the child car seat legislation was high (91/97, 94%). Children aged 2–3 years were less likely reported to be restrained in an age-appropriate car seat than were older children aged 4–5 years (60% versus 95%: χ^2^ = 19.14, *p* < 0.001). Focus group participants highlighted how important their child’s safety was to them, spoke of the influence grandparents had on their use of child car seats and voiced mixed views on the value of authorised child car seat fitters. Future programs should include access to affordable car seats and target community members as well as parents with clear, consistent messages highlighting the safety benefits of using age-appropriate car seats.

## 1. Introduction

Despite legislated road safety measures such as the use of appropriate child car seats [1], road crashes continue to be a leading cause of childhood death and serious injury in Australia. While legislation was shown to have some impact on use of appropriate car seats, research continues to show that not all children are appropriately restrained [2,3]. Over the last decade, Australian studies have reported that only 49–88% of children aged younger than seven years are restrained in the right car seats for their age or size [3,4,5].

Researchers have attempted to gain a clearer understanding of factors that influence child car seat use both in Australia [5,6,7,8,9,10] and elsewhere [11,12,13,14,15]. Age- or size-appropriate child car seat use has been shown to be associated with socio-demographic factors. Age of the child (children aged less than 2 years and children aged more than 9 years) [7,12], parental age (parents aged younger than 35 years) [16], family income (annual household income greater than CAD 40,000 in 2012 [14] or AUD 60,000 in 2010 [5] parents’ educational level (beyond high school) [5,7,14,16], and English being the primary language spoken at home [5] have all been shown to be associated with age-appropriate car seat use. In addition, other factors such as parental knowledge of car seats, [12,14] parenting styles (parents not negotiating with their child about car seat use) [7], and both subjective norms (parents perceived other parents in their community used child car seats) and intention to comply with best practice have also been shown to be associated with use of age-appropriate car seats [11].

In Australia, Aboriginal and Torres Strait Islander people represent 2.8% of the total population [17] yet almost 6% of transport related fatalities [18]. Aboriginal and Torres Strait Islander children aged 0–4 years are more than 4 times more likely to die from a road related injury and more than 2 times more likely to suffer a serious road related injury than other Australians of the same age [18]. While there are numerous studies exploring level of use and factors influencing car seat use in Australia, few have investigated this important issue in a community with a large proportion of Aboriginal families.

This issue has been examined among First Nation populations both in Australia [19,20] and internationally [15,21,22]. Lapidus et al., conducted an observational study of car seat use among Northwest American Indian children from six tribes across three states and found that age-appropriate car seat use was less likely with younger age (OR per year: 0.60; 95% CI 0.48–0.75), front versus rear seating (OR: 0.27; 95% CI 0.16–0.44), and the driver not being the child’s parent (OR: 0.28; 95% CI 0.14–0.58); and more likely if the driver was wearing a seatbelt (OR: 2.39; 95% CI 1.51–3.80). In addition, Lapidus et al. reported low knowledge of appropriate car seat use (49%) and little awareness (47%) of the presence or absence of child car seat legislation in their community [21].

The use of behavioural change theories and models in injury prevention research has increased over the last two decades with researchers recognising the value of applying a theoretical model in both research planning and program development [23,24]. Trifiletti et al. (2005) conducted a review on how behavioural science theories and models were used in unintentional injury prevention research and reported that the PRECEDE-PROCEED theoretical model was the most commonly cited model [23]. Originally developed by Green and Kreuter [25] the PRECEDE-PROCEED model has been widely used as a framework to plan investigation of factors affecting risk taking behaviours, [23,25,26] to plan injury prevention programs [27] and recommended for use in motor vehicle injury control programs [28]. Rivara et al. used the PRECEDE-PROCEED framework to examine the results from focus groups conducted to explore parental attitudes, knowledge and behaviours relating to child booster seat use in Seattle, USA [29]. The authors categorised findings according to the key model constructs: predisposing factors (for example, parents’ attitudes, knowledge and beliefs about booster use); enabling factors (such as access to and availability of resources, having the skills necessary to use the car seats correctly and affordability of car seats); and reinforcing factors (for example, incentives or forms of support or confirmation of how to travel with children from credible sources).

No study has previously reported the use of child car seats and the factors influencing their use among Aboriginal people in Australia despite the high burden of child passenger transport injuries experienced by this population. Consequently, we sought to explore factors affecting age-appropriate child car seat use among preschool children in a regional community with a high proportion of Aboriginal families in New South Wales. Using a mixed methods approach, we applied the PRECEDE-PROCEED theoretical framework to explore parental knowledge and attitudes and self-reported child car seat use. Such information is needed for the development of targeted programs to promote best practice child car seat use in this setting.

## 2. Materials and Methods

Using a mixed-methods approach we conducted a survey (quantitative) and focus groups (qualitative) with parents or carers whose children were aged 3–5 years and were enrolled at one of three early learning centres (two preschools and one long day care centre) in regional New South Wales, Australia.

For the survey, participants were parents and carers whose children, aged 3–5 years, were enrolled in one of the participating early learning centres. The centres were selected based on the number of children attending the centre (more than 20 children aged between 3–5 years), a physical layout that allowed for safe observation of child car seat use (which was needed as part of the pilot program evaluation later in the year), and where at least 20% of the children enrolled at the centres were Aboriginal children.

After obtaining written consent, local trained research assistants interviewed the parents and carers as they arrived at the centre about their knowledge and use of child car seats. Research assistants administered the survey and then parents were invited to fill in their demographic details independently.

The survey was adapted from a previous study [7] to include questions about the current child car seat legislation and Aboriginal status of the children travelling in the car and the parent/carer completing the survey.

Focus group participants were parents whose children aged 3–5 years were enrolled in the participating centres described above. We recruited participants through posters which were displayed at the centres. Participants were also directly approached through personal invitation by the centre director who was asked to encourage parents of Aboriginal children to attend. A researcher experienced in qualitative methods conducted three focus groups of 60–90 min duration at each of the centres, 6–14 weeks after the surveys were administered. We ensured focus groups were conducted during the day at times convenient for parents and centre staff. Parents gave informed written consent and completed a short demographic survey (7 questions) before the discussion began. All focus groups were audio-taped with the consent of participants and transcribed verbatim. Written field notes were made immediately after each focus group by the facilitator.

All survey data was entered onto a secure server using double data entry and errors were corrected. The data was analysed and apart from the demographic data, results are presented using the PRECEDE-PROCEED framework [29,30]. We defined self-reported age-appropriate car seat use as a child aged 2–3 years being in a forward-facing car seat and a child aged 4–5 years being in a booster seat or forward-facing car seat which reflected the NSW legislation. Definitions of child car seats were in accordance with the Australian Standard for child car seats (AS/NZS 1754:2010) [31]. We summarised survey data using descriptive statistics. Univariate analysis of factors associated with age-appropriate car seat use was conducted using chi square tests and where appropriate, Fisher’s exact test. Using backwards elimination, we identified factors independently associated with age-appropriate car seat use, adjusting for clustering at the service level using a marginal effects model with an exchangeable correlation structure. Entry into the model was with a univariate *p*-value of <0.2. A *p* < 0.05 was regarded as statistically significance.

Focus group discussion recordings were listened to in full then transcribed verbatim by a transcription service and then checked for accuracy by the first author. Each transcription was read and re-read and codes were developed deductively, based upon the theoretical framework. Non-verbal communication was not systematically interpreted. STATA version 12 (StataCorp LP, College Station, TX, USA) was used to summarise the quantitative data and NVIVO 9 to analyse the qualitative data.

This study was approved by the Aboriginal Health and Medical Research Council of New South Wales Human Research Ethics Committee (703/09) and the University of Sydney Human Research Ethics Committee (01-2010/12236). It received verbal support from the local Aboriginal Education Consultative Group and was guided by a steering committee comprising representatives from the local Aboriginal community.

## 3. Results

We present the survey results first followed by the focus groups.

### 3.1. Parent/Carer Survey

#### 3.1.1. Survey Participants

Of the 133 families with children turning 3–5 years in 2010 enrolled at the centres, directors reported 44 (33%) were Aboriginal or Torres Strait Islander families. A total of 97 (73%) parents completed the survey. Of those parents, 19 (20%) identified as being of Aboriginal or Torres Strait Islander descent and 30 (31%) children were reported as being of Aboriginal or Torres Strait Islander descent. A majority of families 49 (57%) reported an annual household income of less than AUD 60,000; 11 (11%) participants did not respond to the question regarding household income (Table 1).

#### 3.1.2. Car Seat Use

Overall, use of age-appropriate car seats was reported by 80 (83%) of participants. Parents of children aged 2–3 years were less likely to report their child being restrained in an age-appropriate car seat than were parents of children aged 4–5 years (60% versus 95%: χ^2^ = 19.14, *p* < 0.001). There was no significant difference in reported use of age-appropriate car seats between Aboriginal or Torres Strait Islander children and other children (77% versus 85%: χ^2^ = 1.01, *p* = 0.31).

#### 3.1.3. Predisposing Factors—Awareness and Knowledge

There was a very high level of awareness (*n* = 91; 94%) of the child car seat legislation among survey participants. Of participants who reported age-appropriate car seat use, 74 (93%) were aware of the child car seat legislation, however that awareness was not associated with age-appropriate car seat use (*p* = 0.59). Knowledge of the correct ages for when to move a child from one car seat to the next, according to the legislation, was not as strong. While not all participants responded to the question, 52 (88%) knew that a child must be at least 4 years old to use a booster seat and 67 (91%) knew that a child could legally begin to use an adult seatbelt from 7 years. This knowledge was not associated with age-appropriate use, *p* = 0.16 (Table 2).

Respondents’ knowledge of the safety benefits of the different car seat types for different ages, was associated with age-appropriate car seat use (Table 2). When compared with children not in age-appropriate car seats, respondents with children aged 2–3 years travelling in the right car seat for age were more likely to disagree or strongly disagree with the statement that “A booster seat offers a three-year old child the same level of protection as a forward-facing car seat”. Respondents of children aged 4–5 years were more likely to disagree or strongly disagree with the statement that “An adult seatbelt offers a six-year old the same level of protection as a booster seat.” (χ^2^ = 4.50, *p* = 0.03).

#### 3.1.4. Enabling Factors—Cost of Car Seat and Income

The majority (*n* = 71; 73%) of respondents disagreed or strongly disagreed with the statement that “High cost prevents me from getting the child seat I want to get for my child” and this was not significantly associated with age-appropriate car seat use. Similarly, neither a family’s annual household income (*p* = 0.23) nor the number of children in the family were statistically significantly associated with age-appropriate car seat use (χ^2^ = 0.03, *p* = 0.86).

#### 3.1.5. Reinforcing Factors—Societal Norms

While most survey respondents (*n* = 78; 80%) felt that their child travelled to the early learning centre in the same way other children had travelled, that is similarly restrained, this was not significantly associated with using the right car seat for age (*p* = 0.74), nor was the parent saying that using a car seat was non-negotiable with the child (*p* = 0.63).

While the state road authority was the group people most often cited as who to turn to for information on car seats, this was not associated with being in the right restraint for age. However, receiving information about child car seats from the early childhood centre was statistically associated with age-appropriate car seat use (χ^2^ = 4.20, *p* = 0.04).

In multivariate analysis, only the age of the child was statistically significantly associated with age-appropriate car seat use. The odds of a child using the right car seat for age were higher for children aged 4–5 years compared with children aged 2–3 years (ORadj 10.51, 95% CI: 2.88–38.37). This was indicative of younger children being prematurely graduated into the booster seat (14/35, 40% children aged 2–3 years were reported to be in a booster seat).

### 3.2. Focus Groups

A total of 10 parents or carers participated in the three focus groups. Focus group one had six participants; focus group three had three participants and while three parents indicated interest in the third focus group, only one person participated so this was conducted as a semi-structured interview. All participants were mothers of children aged between 3–5 years enrolled at the centres, spoke English as their primary language at home and in the previous week had travelled with their child by car. Centre directors reported 4 of the 10 parents were parents of Aboriginal children (Table 1).

#### 3.2.1. Predisposing Factors—Awareness and Knowledge

All focus group participants were aware of the new law but their knowledge of the specifics of the law was inconsistent. As with the parents who completed the survey, parents in focus groups indicated that they had a better understanding of the legislation for older children but were less sure of the transition from forward-facing car seat to booster seats than they were of the transition from booster seat to adult seatbelt. While parents were aware that the new legislation focused on the age of the child rather than body weight, there were some parents who disagreed with this approach. Table 3 presents a summary of findings from the focus groups.

Across all groups, parents said that the use of child car seats was essential. Perceptions of risks while travelling with children differed among participants. While all participants reported being concerned about being in a crash with their children, the perception of the likelihood of that event happening differed. Some felt that longer trips were associated with greater risk of a crash while others reported having heard that most crashes happened close to home.

#### 3.2.2. Enabling

In contrast to the survey findings that household income was not associated with car seat use, all focus groups suggested cost could be one of the underlying reasons people thought other parents did not use age-appropriate car seats.

Focus group participants also discussed their experience with, and perceptions of how worthwhile resources and services such as car seat fitters were to using the right car seats, and using them correctly. Some participants felt that using car seats was not difficult, “I just think most of it is common sense”. Discussion about the value of going to an authorised child car seat fitter polarised participants. Some participants described learning from the authorised fitter while others felt paying for an authorised restraint fitter to fit the car seat was a waste of money (Table 3).

#### 3.2.3. Reinforcing

Focus group participants were in agreement that children did not dictate how they travelled in a car and spoke of the active role their children played in reinforcing the use of the right car seat, recounting how their children had reminded them if the parent had forgotten to strap the child in the car seat.

When asked if there were any perceived negatives associated with the use of car seats the responses from focus groups focused on the comfort of the child and ease of use: (when my son is in his car seat) “his legs don’t bend and his legs are dangling and his legs go to sleep.”

The role of grandparents was also explored in the focus groups with participants describing conflicting situations. Some grandparents did not share the same commitment to using car seats while other participants described how some grandparents had been the person driving the use of car seats.

When travelling with peers, some parents voiced reluctance at speaking out about how their child travelled with other people. Others said they were comfortable in insisting that their child use a car seat, giving the welfare of the child as the prime reason.

Further, participants also described pressure from other community members to give lifts when there were not enough car seats for the number of children. They commented that it was a difficult situation:

“I find it hard to say no, and they keep asking, trying to find ways around it when you’ve already said no. You just gotta be blunt and say no.”

In addition, they also described the implications for them if they were to give a lift to a peer’s child not using a car seat including, loss of license (which could then impact their work) or being fined.

Following on with the concept of receiving fines; focus group participants felt that the threat of fines and loss of demerit points helped reinforce the message. Although participants also suggested that if the community does not see the law being enforced then people are more likely to become complacent about following the rules.

## 4. Discussion

This is the first study using both quantitative and qualitative data to report on factors influencing use of age-appropriate child car seats in a community with a large proportion of Australian Aboriginal families. Presented within the PRECEDE-PROCEED framework and combining results from the conduct of surveys with focus groups, we gained important insights regarding the social context of using car seats. We found 80 children (83%) were reported to be restrained in age-appropriate car seats.

For predisposing factors we found that while both the quantitative and qualitative data showed strong awareness and knowledge of the car seat laws, there appeared to be some disconnection between knowing key points of the legislation and knowledge of the safety benefits associated with it, particularly around the ages of transition [8,10,32]. The strong association between knowledge and use of age-appropriate car seats is well documented [7,14] and our findings support the need to continue to deliver programs that clearly define the transition points and also focus on the relevant safety benefits associated with using the right car seat.

Age of the child was a key enabling factor. Our findings that children aged 4–5 years were more likely to be restrained in an age-appropriate car seat than children aged 2–3 years, are comparable to previous studies conducted in Australia [4,5,10]. Keay et al. in a survey in the same calendar year as this study reported 54% of children aged 2–3 years versus 88% of children aged 4–5 years were restrained in an age-appropriate car seat [5].

We found the number of children in the family was not significantly associated with age-appropriate car seat use. This finding is in contrast to earlier studies reporting the more children in the family the less likely the child would be appropriately restrained [5,7]. These findings are comparable to those of Keay et al. who reported age-appropriate car seat use was significantly associated with number of children in the family among children aged 2–3 years, however it was not a factor for age-appropriate car seat use among children aged 4–5 years [5].

Another key enabling factor is affordability. The impacts of household income and perception of cost on child car seat use is complex. Despite other authors concluding low household income was associated with suboptimal car seat use [5,14,16], studies have also found that parents reported cost was not a barrier [10]. In many qualitative studies, however, the cost of a child car seat is often cited as a barrier to child car seat use [8,29,33,34]. In this study, cost was not perceived to be an issue for either survey or focus group participants to travel with their children in the right car seat and similarly household income was not associated with child car seat use in the survey, yet focus group participants felt cost could be an issue for other parents warrants further exploration. It is possible, however, that participants’ perception about other parents’ motivations and determinants of car seat use in this study could be explained through ‘third person effect’ (where a person believes an effect or issue is greater for others than for themselves) [35].

Skills development may also be regarded as an enabling factor. While studies that include an element of ‘hands-on’ education [36] have been shown to be effective, how that education is delivered may be critical for this community. If authorised car seat fitters are included in the program then the concerns voiced by focus group participants about car seat fitters (such as cost of the service) should be addressed. For example, a child car seat education program could also include access to free car seat checks and free vehicle adjustments required to correctly install a car seat. Such interventions have been shown to be effective elsewhere [37,38].

Approaches that target factors that communities identify as reinforcing car seat use should also be considered in program development. This includes information provided at centres and enforcement of car seat legislation that addresses how parents can manage situations when family members and friends do not support appropriate child car seat use. Therefore, these results could inform development of more targeted programs, such as involving health and community workers in the delivery of programs to reinforce core messages.

Similarly, the qualitative data highlighted the complex role of extended family in reinforcing, and simultaneously challenging appropriate child car seat use. In particular, the key role grandparents were reported to play in influencing car seat use indicates potential benefit in developing programs specifically targeting these individuals. This has been shown to be effective in other programs elsewhere. For example, involving grandparents in motivational counselling and family support targeting mental health among Australian Aboriginal families [39] and in increasing youth sport participation among Canadian First Nation families [40].

There are some limitations to this study. Resource limitations meant the survey was not well powered. A lack of statistically significant results in the logistic regression analysis (and the wide confidence intervals) could be explained by the small sample size. In addition, the proportion of children travelling in the right seat for their age is likely an overestimate due to possible bias in reporting. These survey results are based on parents’ and carers’ reporting of their use; it is possible observational studies would identify fewer children travelling in the right car seat for their age and size [41]. Further, the focus groups involved 10 participants in total and as we had limited resources, we were unable to conduct more focus groups to ensure saturation had been reached. These findings, therefore should not be generalised across broader populations and regions. Results from this study will inform a larger state-wide study.

It is a strength of this study that results were combined using data from both the survey (quantitative data) and focus groups (qualitative data), allowing a broader interpretation of factors affecting age-appropriate child car seat use.

While this community was not a discrete Aboriginal community, the group does represent a community with a greater proportion of Aboriginal families (20%), and children (31%), compared with Australia as a whole where 2.8% of the population identifies as Aboriginal or Torres Strait Islander. We recognise that all Aboriginal communities are different and that any community-based program will need to be tailored to suit the specific community, however, findings from this study can inform future child car seat programs. Importantly, key findings from this pilot study, notably the need to focus on safety as part of the core messaging and inclusion of hands-on support has informed the planning and development of a state-wide child car seat program currently being evaluated across 12 Aboriginal communities in New South Wales.

## 5. Conclusions

This study builds on previous work conducted in regional communities by Stewart et al. (2007) [10] and provides valuable insight into determinants of car seat use among low income parents in regional New South Wales, where a significant proportion of the community are Aboriginal families. Findings from this explorative research indicate that programs targeting optimal car seat use should contain clear, consistent messages that focus on the safety benefits associated with using the right car seat for age. Program development needs to be mindful of affordability and if experts such as authorised car seat fitters are included then it should be done in a way that is accessible and builds local capacity and expertise within the community. To reinforce messages, programs should target grandparents as well as parents and should include provision of information through trusted sources such as preschools. The focus of the messages should contain clear information about the ages and car seat types and transitions and, importantly, that messaging should focus on the safety benefits of being in the right seat for the child’s age and size. Finally, program components could address child passenger safety in alternate transport options for those parents who lack access to a car.

## Figures and Tables

**Table 1:** Age, educational attainment and family characteristics of focus group (*n* = 10) and survey participants (*n* = 97).

Characteristics	Focus Group Total *n* (%) *n* = 10	Survey Total *n* (%) *n* = 97	Survey Respondents Whose Children Were Aboriginal *n* (%) *n* = 30
**Participants’ Age Group**			
18–25 years	3 (30)	12 (12)	6 (20)
26–35 years	5 (50)	48 (50)	16 (53)
36–45 years	2 (20)	31 (32)	6 (20)
Older than 45 years	0 (0)	6 (6)	2 (7)
**Participants’ Highest Level of Educational Attainment**			
Some secondary school or less	2 (20)	27 (28)	12 (40)
Completed secondary school	3 (30)	18 (19)	7 (23)
Some tertiary (university or TAFE)	3 (30)	30 (31)	9 (30)
Completed tertiary	2 (20)	22 (23)	2 (7)
**Family Characteristics ^1^**			
Annual household income less than AUD 60,000 p.a.		49 (57) ^2^	20 (80) ^3^
Children aged 4–5 years		62 (64)	21 (70)
Less than 3 children aged younger than 18 years in the household		52 (54)	12 (40)

^1^ Questions asked of survey participants only; ^2^ 11 missing data; ^3^ 5 missing data.

**Table 2:** Comparison of predisposing, enabling and reinforcing factors for car seat use between those reporting car seat use assessed to be age-appropriate and those reporting car seat use assessed to be inappropriate, for 97 children aged 2–5 years.

Factors	Appropriate Car Seat Use *n* = 80, *n* (%)	Inappropriate Car Seat Use *n* = 17, *n* (%)	*p*
**Predisposing Factors—Awareness and Knowledge**			
Aware that a new child car seat law was being introduced in the year the study was conducted.	74 (93)	17 (100)	0.59 ^1^
Responded correctly to the questions: “According to the law up to what age must a child use a forward-facing car seat and a booster seat.” ^2^	54 (95)	8 (80)	0.16 ^1^
Knowledge of protective benefits of booster seat over adult belt and forward-facing car seat over a booster seat. ^3^	69 (86)	11 (65)	0.03
**Enabling Factors—Cost and Income**			
Age of the child—child is aged 4–5 years	59 (74)	3 (18)	<0.001 ^1^
Cost does NOT prevent parent from purchasing the car seat they want to	60 (75)	11 (65)	0.38
Annual household income is at least $AUD60,000	33 (47)	4 (27)	0.23 ^1^
Only 1 or 2 children in family	37 (46)	7 (44)	0.86
Type of seat is not negotiable with the child	15 (88)	74 (93)	0.63 ^1^
**Reinforcing Factors—Peer Norms and Support**			
Agree or strongly agree with the statement that my child is in the same car seat as other children the same age	65 (81)	13 (77)	0.74 ^1^
Information provided at the service	30 (38)	2 (12)	0.04

^1^ Fisher’s exact test; ^2^ Marked correct if parents of children aged 2–3 years responded to the question: “According to the law up to what age must a child use a forward-facing car seat?” with “up to 4 years”; and if parents of children aged 4–5 years responded to the question: “According to the law up to what age must a child use a booster seat?” with “up to 7 years”; ^3^ Marked correct if parents of children aged 2–3 years disagreed or strongly disagreed to the statement: “A booster seat offers a three year old child the same protection as a forward-facing car seat” and if parents of children aged 4–5 years disagreed or strongly disagreed to the statement: “An adult seatbelt offers a six year old child the same protection as a booster seat”.

**Table 3:** PRECEDE-PROCEED framework applied to describe factors affecting car seat use reported by parents in focus groups.

**Predisposing Factors (Awareness and Knowledge of the Legislation, Perceptions of Risk)**
Confusion about the legislation:
“It is really difficult like you’ve got to read through it like several times before you can actually work out what the rules are.”
Disagreement with legislation on how to know which car seat to use:
“I’ll just go by the size of the kid and how well it fits in the seat.” “Should be done on weight.”
Parents suggested a perception of low risk to explain others not using a car seat:
“‘I’m only ducking down the road or ducking to the corner shop’.” “They don’t think they’re gonna end in an accident or something like that. Some people just don’t realise until it’s too late. They think ‘Oh it’s not going to be me, I’m not going to get involved in an accident; it’s going to be alright. I’ll just shove the kid in ‘cos I’m in a hurry and just go.’”
**Enabling Factors (Affordability and Ease of Use)**
Perception of high cost of car seats:
“I think maybe some parents just don’t care, don’t know, or have lots of kids and can’t afford it—think money is the main barrier.” “…they can’t afford to go and buy another booster.”
Perception of value of car seat fitter (negative):
“Apart from being charged an absolute fortune…when I’m down at nanny and granddads’ giving them my car seat taking it out thinking ‘well that was just a waste of…I’m not going back to him to spend that amount of money every time I need to take the car seat in and out of the car it’s totally impractical!”
Perception of the value of car seat fitter (positive):
“Well when we got the first one fitted, G (husband) got in the boot with him and he ran him through it.”
**Reinforcing Factors (Perception of Enforcement, Peer Influence)**
Pressure reported from other peers:
“And they say ‘Oh please it’s just up at the shop’, you know trying to con me and I say ‘No. I’m not getting a fine’. And if we have an accident. [Pause] They don’t understand, they don’t own a car they don’t have a license themselves so it doesn’t really bother them that you could lose your license, you get a fine and that their child gets hurt.”
Lack of perception of enforcement:
“Well, (parents could think) no one’s been fined, nothing’s happened, I’m only ducking down the road or ducking to the corner shop, its fine. I’ll just stick them in the front seat—it doesn’t matter. Or it’s in my husband’s car and I need to go get milk, so I’ll just pop them in the front seat.”
Safety of child over-rides peer pressure to not travel safely:
“I wouldn’t let him in the car [without a car seat] ‘cos he’s the number one thing in my world.’”
Responsibility for other children:
“We have rules in my car and he (another child) had to repeat them to me.”
Strong influence of others in the family:
“The first thing my mother said was ‘Oh, you better ring her back just to see if she had a seat for the baby’.” “…she’ll come over and say ‘Nup, you need a seat before you hop into my car.’ Feel I suppose she’s right.” “A few times my mum has had to pick me up from somewhere and she’s come up and refused to take me if I didn’t have a seat,” says a mother of 3 children. Both she and her partner are currently learning to drive.
Role of parents influencing grandparents:
“We made sure that Nanny knew what the go was and makes sure that Poppy doesn’t cut any corners. We showed it on ours [car] and said it’s really important.”
